# Cardiac disease in patients with inflammatory myopathy

**DOI:** 10.1093/eschf/xvag019

**Published:** 2026-01-13

**Authors:** Leonhard Binzenhöfer, Katharina Strauß, Linus Seifert, Inas Saleh, Michael Czihal, Hendrik Schulze-Koops, Enzo Lüsebrink

**Affiliations:** Medizinische Klinik und Poliklinik I, Klinikum der Universität München, München, Germany; DZHK (German Center for Cardiovascular Research), partner site Munich Heart Alliance, München, Germany; Medizinische Klinik und Poliklinik I, Klinikum der Universität München, München, Germany; DZHK (German Center for Cardiovascular Research), partner site Munich Heart Alliance, München, Germany; Medizinische Klinik und Poliklinik I, Klinikum der Universität München, München, Germany; DZHK (German Center for Cardiovascular Research), partner site Munich Heart Alliance, München, Germany; Medizinische Klinik und Poliklinik I, Klinikum der Universität München, München, Germany; DZHK (German Center for Cardiovascular Research), partner site Munich Heart Alliance, München, Germany; Sektion Angiologie, Medizinische Klinik und Poliklinik IV, Klinikum der Universität München, München, Germany; Sektion Rheumatologie und Klinische Immunologie, Medizinische Klinik und Poliklinik IV, Klinikum der Universität München, Pettenkoferstraße 8a, 80336 München, Germany; Medizinische Klinik und Poliklinik II, Universitätsklinikum Bonn, Venusberg-Campus 1, 53127 Bonn, Germany

**Keywords:** Inflammatory myopathy, Structural heart disease, Cardiac arrhythmia

## Abstract

**Background and Aims:**

Inflammatory myopathies (IMs) are a group of autoimmune diseases characterized by progressive symmetric muscle weakness and various extramuscular manifestations. Cardiac involvement in IM has been associated with worse outcomes, but evidence to support specific screening and management algorithms for cardiac comorbidities in IM is still limited.

**Methods:**

For this observational study, 37 adult IM patients recruited from the rheumatology outpatient clinic at Ludwig-Maximilians-University Hospital between August 2023 and January 2025 completed a questionnaire on IM characteristics, management, clinical events, and cardiac disease. Self-reported data were verified and complemented using clinical records. The main study endpoints included the incidence of cardiomyopathy, documented cardiac arrhythmias, and myocardial infarction following the diagnosis of IM.

**Results:**

The median age at last follow-up was 60 years and 35.1% were male. The most common cardiac symptoms reported by study participants included dyspnoea (45.9%), congestion/oedema (40.5%), palpitations (32.4%), and chest pain (18.9%). Arterial hypertension was diagnosed in 27% after IM had been established. Supraventricular and ventricular arrhythmias were documented in 10.8% and 5.4%, respectively. Echocardiography was performed in all study participants, revealing left ventricular diastolic dysfunction in 35.1%. Severe structural heart disease and cardiac adverse events, including acute myocardial infarction, severe valvular disease, and left ventricular systolic dysfunction, were documented only in isolated cases of IM.

**Conclusions:**

Cardiac symptoms, risk factors, and structural abnormalities are prevalent in a substantial proportion of patients with IM. Routine cardiologic assessment, including echocardiography, may be advisable. Further evidence from prospective longitudinal studies is needed to optimize screening algorithms and multidisciplinary management.

## Background

Inflammatory myopathies (IMs) are a group of autoimmune diseases that include dermatomyositis, anti-synthetase syndrome, polymyositis, inclusion body myositis, immune-mediated necrotizing myopathy, and overlap myositis.^1,2^ The common clinical feature is progressive symmetric proximal muscle weakness, though various extramuscular tissues may also be involved, such as the skin, joints, lungs, or heart.^1,3^ Cardiac involvement in IM may present as structural heart disease with abnormalities on echocardiography or cardiac magnetic resonance (CMR) imaging, as well as rhythm and conduction disturbances.^4–6^ Several population-based studies from different regions have suggested that cardiac complications are associated with higher mortality in IM.^7–9^ However, there is no standardized definition of cardiac involvement, and previous analyses have employed varying approaches to assess its association with outcomes. In addition, the prevalence and clinical significance of subclinical cardiac disease in patients with IM remain uncertain. According to the 2021 European Alliance of Associations for Rheumatology (EULAR) guidelines, cardiovascular risk management in patients with IM should adhere to recommendations employed in general populations, but evidence to support specific screening and management algorithms for cardiac comorbidities in IM is still limited.^10^

## Aims

The aim of the present investigation was to provide a comprehensive analysis of cardiac symptoms, incident cardiac comorbidities, and cardiologic management in a large unselective cohort of IM patients with obligate systematic echocardiography to inform screening and management algorithms.

## Methods

Patients were recruited from the rheumatology department at Ludwig-Maximilians-University Hospital between August 2023 and January 2025. All study participants provided written informed consent. Adult patients (≥18 years) diagnosed with IM according to the 2017 EULAR/American College of Rheumatology diagnostic criteria were eligible for inclusion.^3^ Patients were excluded, if IM was not confirmed or if patients were unable to complete the study questionnaire or were last seen in the outpatient clinic prior to January 2022. The study questionnaire consisted of questions regarding the characteristics and management of IM, clinical events, and cardiac disease and management. Self-reported data were verified and complemented using clinical records. Standard definitions were used to define the parameters of interest. Left ventricular (LV) systolic dysfunction was defined as LV ejection fraction <50%. Right ventricular (RV) dysfunction was defined as tricuspid annular plane systolic excursion <18 mm and/or fractional area change ≤35% and/or visual impairment of RV contractility. Chronic kidney failure was defined as Kidney Disease Improving Global Outcomes Stage G3a or worse. The main study endpoints included the incidence of cardiomyopathy, documented cardiac arrhythmias, and myocardial infarction following the diagnosis of IM. Secondary endpoints included the emergence of new cardiac symptoms, the onset of incident arterial hypertension, the presence of structural abnormalities other than cardiomyopathy, cardiac procedures, and pharmacological management.

## Results

**Figure 1:**
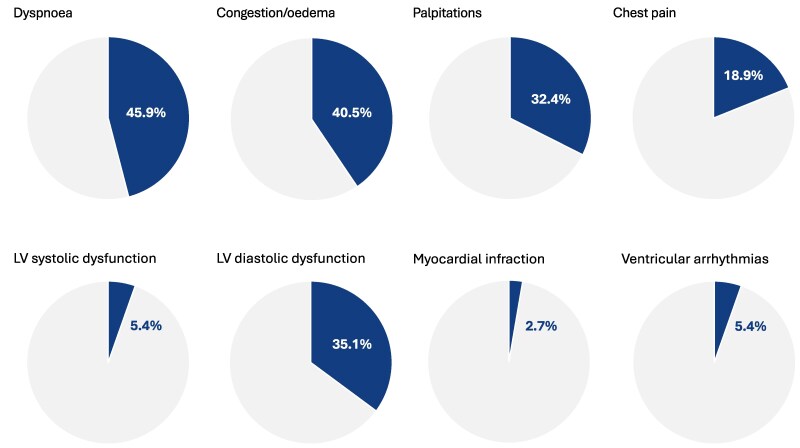
Cardiac symptoms, echocardiographic findings, and complications in 37 inflammatory myopathy patients. LV, left ventricular

A total of 56 patients diagnosed with myositis were screened for study participation, of whom 13 did not meet eligibility criteria, and 6 patients were excluded because they had died (*n* = 3), had missing contact information (*n* = 2), or declined participation (*n* = 1). Overall, 37 patients completed the study questionnaire and were included in the analysis. The median age at last follow-up was 60 years, and 35.1% were male (*Table 1*). Inflammatory myopathy was diagnosed at a median age of 52 years and was confirmed within the first year after symptom onset in 73%. Severe non-cardiac complications, such as stroke, end-stage kidney failure, or arterial occlusion requiring intervention, occurred overall rarely. More frequent organ manifestations included cutaneous involvement in 70.3% and interstitial lung disease in 51.4% of cases. Inflammatory myopathy was classified as dermatomyositis in 35.1%, polymyositis in 18.9%, and anti-synthetase syndrome in 29.7%. The majority of patients were current or former smokers, whereas other cardiovascular risk factors were less common (*Table 2*). The most common cardiac symptoms after IM diagnosis were dyspnoea in 45.9%, chest pain in 18.9%, and signs of congestion/oedema in 40.5% (*Figure 1*). Additionally, 32.4% of patients reported palpitations, although supraventricular and ventricular arrhythmias were documented in only 10.8% and 5.4%, respectively. Arterial hypertension was diagnosed in 27% after IM had been established. Echocardiography was performed in all study participants. The most prevalent finding was LV diastolic dysfunction in 35.1%, followed by moderate or severe mitral valve regurgitation in 8.1%. LV and RV systolic dysfunction was noted in 5.4%, respectively. It is noteworthy that only one patient suffered acute myocardial infarction after IM diagnosis. Almost all patients received immunosuppressive therapy with temporary glucocorticoids (94.6%) and a disease-modifying anti-rheumatic drug (DMARD; *Table 3*). Coronary angiography was performed in 11 patients, of whom 4 were diagnosed with coronary artery disease. However, none of the patients required percutaneous coronary intervention or coronary artery bypass grafting.

**Table 1:** Demographics, disease characteristics, diagnosis, and complications/non-cardiac organ manifestations of inflammatory myopathy

	IM (*n* = 37)
Demographics	
Age at last follow-up (years), median (IQR)	60 (52–70)
Sex (male), *n* (%)	13 (35.1)
IM characteristics	
Dermatomyositis, *n* (%)	13 (35.1)
Polymyositis, *n* (%)	7 (18.9)
Anti-synthetase syndrome, *n* (%)	11 (29.7)
Other/non-specific, *n* (%)	6 (16.2)
Acute disease onset, *n* (%)	10 (27.0)
Chronic disease onset, *n* (%)	22 (59.5)
Major relapse, *n* (%)	11 (29.7)
Diagnosis of IM	
Age at diagnosis (years), median (IQR)	52 (40–65)
BMI at diagnosis (kg/m^2^), median (IQR)	23.71 (21.60–26.70)
Number of physician visits until establishment of definite diagnosis (*n*), median (IQR)	4.00 (2.00–8.25)
Objective symmetric weakness of the proximal upper extremities, *n* (%)	15 (40.5)
Objective symmetric weakness of the proximal lower extremities, *n* (%)	14 (37.8)
Neck flexors relatively weaker than neck extensors, *n* (%)	3 (8.1)
Proximal leg muscles relatively weaker than distal muscles, *n* (%)	14 (37.8)
Heliotrope rash, *n* (%)	2 (5.4)
Gottron's papules, *n* (%)	4 (10.8)
Gottron's sign, *n* (%)	3 (8.1)
Dysphagia or oesophageal dysmotility, *n* (%)	5 (13.5)
Elevated serum levels of creatine kinase or lactate dehydrogenase or aspartate aminotransferase (ASAT/AST/SGOT) or alanine aminotransferase (ALAT/ALT/SGPT), *n* (%)	20 (54.1)
Modified Rankin scale, median (IQR)	2 (1–2)
Medical Research Council scale, median (IQR)	
Shoulder abductors	5 (4–5)
Elbow flexors	5 (5–5)
Wrist extensors	5 (5–5)
Hip flexors	5 (4–5)
Knee extensors	5 (4–5)
Foot dorsiflexors	5 (5–5)
B-symptoms at IM diagnosis	
Fever, *n* (%)	14 (37.8)
Arthralgias, *n* (%)	31 (83.8)
Sicca, *n* (%)	13 (35.1)
Night sweats, *n* (%)	14 (37.8)
Myalgia, *n* (%)	28 (75.7)
Raynaud, *n* (%)	21 (56.8)
Weight loss, *n* (%)	16 (43.2)
Hair loss, *n* (%)	8 (21.6)
Bleeding tendency (excluding drug related), *n* (%)	2 (5.4)
Antibodies	
Anti-Jo-1, *n* (%)	11 (29.7)
Anti-PL-12, *n* (%)	2 (5.4)
Anti-SRP, *n* (%)	0 (0.0)
Anti-Mi-2, *n* (%)	2 (5.4)
Anti-MDA5, *n* (%)	3 (8.1)
Anti-NXP-2, *n* (%)	1 (2.7)
Anti-TIF-1γ, *n* (%)	1 (2.7)
Anti-SAE, *n* (%)	2 (5.4)
Anti-HMGCR, *n* (%)	1 (2.7)
Anti-Ro/SSA, *n* (%)	17 (45.9)
Anti-La/SSB, *n* (%)	1 (2.7)
Anti-Sm, *n* (%)	3 (8.1)
Anti-ribonucleoprotein, *n* (%)	4 (10.8)
Detection of 1 IM-associated/-specific antibody, *n* (%)	10 (27.0)
Detection of 2 IM-associated/-specific antibodies, *n* (%)	13 (35.1)
Detection of 3 IM-associated/-specific antibodies, *n* (%)	4 (10.8)
Muscle biopsy findings	
Muscle biopsy performed, *n* (%)	10 (27.0)
Muscle infarcts, perifascicular atrophy, capillary necrosis, *n* (%)	3 (8.1)
CD8^+^ T cells invading MHC-I-expressing muscle fibres, *n* (%)	0 (0)
Myophagocytosis, *n* (%)	1 (2.7)
Unspecific, *n* (%)	3 (8.1)
Complications and non-cardiac organ manifestations	
Stroke/transitory ischaemic attack, *n* (%)	2 (5.4)
Permanent disability requiring constant nursing care, *n* (%)	2 (5.4)
End-stage kidney disease requiring dialysis/kidney transplantation, *n* (%)	0 (0.0)
Arterial occlusion requiring intervention/critical limb ischaemia/amputation, *n* (%)	1 (2.7)
Cutaneous involvement, *n* (%)	26 (70.3)
Lymphedema, *n* (%)	4 (10.8)
Interstitial lung disease, *n* (%)	19 (51.4)
Oesophageal involvement, *n* (%)	9 (24.3)
Malignancy, *n* (%)	0 (0.0)
Interval between first physician contact related to IM symptoms and confirmed diagnosis	
≤1 year, *n* (%)	27 (73.0)
1–2 years, *n* (%)	2 (5.4)
2–3 years, *n* (%)	1 (2.7)
3–4 years, *n* (%)	1 (2.7)
4–5 years, *n* (%)	2 (5.4)
>5 years, *n* (%)	2 (5.4)
Medical specialty initially suspecting IM	
Rheumatology, *n* (%)	14 (37.8)
Cardiology, *n* (%)	0 (0.0)
Angiology, *n* (%)	2 (5.4)
Dermatology, *n* (%)	4 (10.8)
Ophthalmology, *n* (%)	0 (0.0)
Neurology, *n* (%)	0 (0.0)
Orthopaedics, *n* (%)	1 (2.7)
Otorhinolaryngology, *n* (%)	1 (2.7)
Pulmonology, *n* (%)	4 (10.8)
General medicine, *n* (%)	9 (24.3)
Emergency medicine, *n* (%)	5 (13.5)
Other, *n* (%)	3 (8.1)

BMI, body mass index; IM, inflammatory myopathy; IQR, interquartile range; MHC-1, major histocompatibility complex 1.

**Table 2:** Cardiovascular symptoms and diagnostic findings after inflammatory myopathy diagnosis

	IM (*n* = 37)
Cardiopulmonary comorbidities prior to IM diagnosis	
Cardiomyopathy, *n* (%)	0 (0.0)
Congestive heart failure, *n* (%)	0 (0.0)
Coronary artery disease, *n* (%)	0 (0.0)
Previous myocardial infarction, *n* (%)	0 (0.0)
Previous coronary angiography, *n* (%)	2 (5.4)
Previous percutaneous coronary intervention, *n* (%)	0 (0.0)
Previous vascular intervention other than coronary, *n* (%)	0 (0.0)
Chronic kidney disease, *n* (%)	2 (5.4)
Chronic pulmonary disease, *n* (%)	6 (16.2)
Chronic inflammatory disease other than rheumatic disease, *n* (%)	6 (16.2)
Cardiovascular risk factors	
Diabetes mellitus, *n* (%)	5 (13.5)
Arterial hypertension, *n* (%)	6 (16.2)
Dyslipidaemia, *n* (%)	8 (21.6)
Smoking history or active smoker, *n* (%)	20 (54.1)
Active smoker at time of diagnosis, *n* (%)	8 (40.0)
Pack years, median (IQR)	8.25 (1.00–16.25)
Family history of cardiovascular disease, *n* (%)	10 (27.0)
Cardiac symptoms after IM diagnosis	
Dyspnoea, *n* (%)	17 (45.9)
Chest pain, *n* (%)	7 (18.9)
Congestion/oedema, *n* (%)	15 (40.5)
Palpitations, *n* (%)	12 (32.4)
Syncope, *n* (%)	2 (5.4)
Laboratory findings	
Peak creatine kinase myocardial band (U/l), median (IQR)	58 (21.25–124)
Peak high-sensitivity troponin T (ng/ml), median (IQR)	0.034 (0.013–0.319)
New onset arterial hypertension after IM diagnosis	
New onset arterial hypertension requiring medical therapy after diagnosis of IM, *n* (%)	10 (27.0)
Arrhythmias after IM diagnosis	
Conduction disorders, *n* (%)	5 (13.5)
Ventricular arrhythmias, *n* (%)	2 (5.4)
Supraventricular arrhythmias, *n* (%)	4 (10.8)
New structural abnormalities and biopsy findings after IM diagnosis	
Echocardiography performed, *n* (%)	37 (100.0)
Left ventricular systolic dysfunction, *n* (%)	2 (5.4)
Regional wall motion abnormalities, *n* (%)	2 (5.4)
Left ventricular diastolic dysfunction, *n* (%)	13 (35.1)
Right ventricular systolic dysfunction, *n* (%)	2 (5.4)
Moderate/severe aortic valve regurgitation, *n* (%)	1 (2.7)
Moderate/severe aortic valve stenosis, *n* (%)	1 (2.7)
Moderate/severe mitral valve regurgitation, *n* (%)	3 (8.1)
Pericardial effusion, *n* (%)	1 (2.7)
Tricuspid regurgitation gradient >31 mmHg, *n* (%)	2 (5.4)
Cardiac magnetic resonance imaging performed, *n* (%)	9 (24.3)
Cardiomyopathy, *n* (%)	0 (0.0)
Endomyocardial biopsy performed, *n* (%)	1 (2.7)
Signs of inflammation, *n* (%)	1 (100.0)
Intracardiac thrombus, *n* (%)	0 (0.0)
Myocardial ischaemia after IM diagnosis	
Electrocardiographic signs of myocardial ischaemia, *n* (%)	2 (5.4)
Myocardial infarction, *n* (%)	1 (2.7)

IM, inflammatory myopathy; IQR, interquartile range.

**Table 3:** Management of inflammatory myopathy and cardiac disease

	IM (*n* = 37)
Management of IM	
Hospital admission for IM treatment, *n* (%)	21 (56.8)
Glucocorticosteroids for initial immunosuppressive therapy, *n* (%)	35 (94.6)
Oral application of glucocorticosteroids *n* (%)	33 (94.3)
Intravenous application of glucocorticosteroids, *n* (%)	13 (37.1)
Long-term immunosuppressive medication before IM diagnosis, *n* (%)	5 (13.5)
Adjunctive immunomodulatory therapy, *n* (%)	36 (97.3)
Conventional synthetic DMARDs	
Azathioprine, *n* (%)	20 (54.1)
Methotrexate, *n* (%)	27 (73.0)
Mycophenolate, *n* (%)	11 (29.7)
Cyclophosphamide, *n* (%)	8 (21.6)
Hydroxychloroquine, *n* (%)	5 (13.5)
Cyclosporin A, *n* (%)	1 (2.7)
Leflunomide, *n* (%)	2 (5.4)
Biologic DMARDs	
Rituximab, *n* (%)	9 (24.3)
IL-6 receptor antagonists, *n* (%)	2 (5.4)
If yes, Tocilizumab, *n* (%)	2 (100.0)
If yes, Sarilumab, *n* (%)	0 (0.0)
Abatacept, *n* (%)	1 (2.7)
Targeted specific DMARDs	
Janus kinase inhibitors, *n* (%)	2 (5.4)
If yes, Baricitinib, *n* (%)	2 (100.0)
If yes, Tofacitinib, *n* (%)	0 (0.0)
If yes, Upadacitinib, *n* (%)	1 (50.0)
Other immunomodulatory medications	
Intravenous immune globulin, *n* (%)	15 (40.5)
Cardiac procedures	
Coronary angiography performed, *n* (%)	11 (29.7)
Coronary artery disease without indication for revascularization, *n* (%)	4 (36.4)
Percutaneous coronary intervention, *n* (%)	0 (0.0)
Coronary lesions other than atherosclerotic, *n* (%)	1 (9.1)
Coronary artery bypass grafting, *n* (%)	0 (0.0)
Surgical/interventional valvular procedure, *n* (%)	1 (2.7)
Pacemaker implantation after IM diagnosis, *n* (%)	1 (2.7)
ICD implantation after IM diagnosis, *n* (%)	0 (0.0)
Cardiovascular medication	
ASA, *n* (%)	8 (21.6)
Initiated after IM diagnosis, *n* (%)	8 (100.0)
P2Y12 inhibitor, *n* (%)	0 (0.0)
Initiated after IM diagnosis, *n* (%)	
Dual antiplatelet therapy, *n* (%)	0 (0.0)
Initiated after IM diagnosis, *n* (%)	
NOAC, *n* (%)	4 (10.8)
Initiated after IM diagnosis, *n* (%)	2 (50.0)
Vitamin K antagonist, *n* (%)	2 (5.4)
Initiated after IM diagnosis, *n* (%)	1 (50.0)
Beta-blocker, *n* (%)	15 (40.5)
Initiated after IM diagnosis, *n* (%)	9 (60.0)
Diuretics, *n* (%)	10 (27.0)
Initiated after IM diagnosis, *n* (%)	9 (90.0)
Aldosterone antagonist, *n* (%)	1 (2.7)
Initiated after IM diagnosis, *n* (%)	1 (100.0)
ARNI, *n* (%)	0 (0.0)
Initiated after IM diagnosis, *n* (%)	
SGLT2 inhibitor, *n* (%)	2 (5.4)
Initiated after IM diagnosis, *n* (%)	2 (100.0)
ACE-inhibitor/AT1-receptor antagonist, *n* (%)	12 (32.4)
Initiated after IM diagnosis, *n* (%)	10 (83.3)
Statin, *n* (%)	12 (32.4)
Initiated after IM diagnosis, *n* (%)	11 (91.7)
Calcium antagonist, *n* (%)	10 (27.0)
Initiated after IM diagnosis, *n* (%)	10 (100.0)
Antiarrhythmic medication (other than beta-blocker), *n* (%)	3 (8.1)
Initiated after IM diagnosis, *n* (%)	2 (66.7)

ACE, angiotensin converting enzyme; ARNI, angiotensin receptor neprilysin inhibitor; ASA, acetyl-salicylic acid; DMARD, disease-modifying anti-rheumatic drug; ICD, implantable cardioverter defibrillator; IL, interleukin; IM, inflammatory myopathy; NOAC, novel oral anticoagulant; SGLT, sodium-dependent glucose co-transporter.

## Conclusions

In this observational study, severe cardiac comorbidities, including acute myocardial infarction, LV systolic dysfunction, and ventricular arrhythmias, were documented only in isolated cases of IM during the follow-up of eight years. Previous analyses have found a higher prevalence of coronary artery disease among IM patients, which may be driven by a combination of common risk factors and inflammation-induced atherosclerosis.^6,11,12^ Interestingly, Tisseverasinghe *et al*.^13^ found an inverse correlation between treatment with conventional synthetic DMARDs and risk of myocardial infarction or stroke in IM patients. Although we did not directly investigate markers of inflammatory activity, the overall low frequency of such complications in our study supports the hypothesis that immunomodulatory combination therapy may limit the risk of major cardiovascular adverse events. However, the 27% incidence of arterial hypertension after IM diagnosis, along with the relatively high rate of diabetes and nicotine use at baseline, also emphasizes the need for screening and optimization of traditional cardiovascular risk factors. Another notable finding was the presence of LV diastolic dysfunction in approximately one-third of our study population. In this context, dyspnoea and signs of congestion, reported by >40% of patients, may represent clinical correlates of heart failure with preserved ejection fraction. Several previous analyses found that diastolic dysfunction is common among patients with IM and that echocardiographic parameters may correlate with disease duration.^4,14,15^ In a meta-analysis of 13 studies by Zhang *et al*.,^14^ mitral annular early diastolic velocity and peak of early/late diastolic flow velocity ratio were both decreased in adult IM patients compared with controls. Some authors have concluded that LV diastolic dysfunction may represent early cardiac involvement in IM, suggesting a potential benefit of routine screening for subclinical abnormalities by electrocardiography, echocardiography, or even CMR.^6^ Main limitations of our analysis include survivor bias, information bias, misclassification bias, and other biases related to retrospective data collection as well as potential centre bias, which may have led to a higher number of polymyositis cases as epidemiologically expected. Further evidence is needed to better understand the pathophysiology of cardiac involvement, the progression of structural and arrhythmic heart disease in relation to inflammatory disease activity, and the optimal management strategies to improve multidisciplinary care for these complex patients.

## Declarations

### Data Availability

The data are not publicly available due to ethical restrictions and legal constraints. Readers may contact the corresponding authors for reasonable requests for the data. De-identified data may be provided after approval from the ethical review board.
